# Two Different Therapeutic Approaches for SARS-CoV-2 in hiPSCs-Derived Lung Organoids

**DOI:** 10.3390/cells11071235

**Published:** 2022-04-05

**Authors:** Paola Spitalieri, Federica Centofanti, Michela Murdocca, Maria Giovanna Scioli, Andrea Latini, Silvia Di Cesare, Gennaro Citro, Antonio Rossi, Augusto Orlandi, Shane Miersch, Sachdev S. Sidhu, Pier Paolo Pandolfi, Annalisa Botta, Federica Sangiuolo, Giuseppe Novelli

**Affiliations:** 1 Department of Biomedicine and Prevention, University of Rome Tor Vergata, Via Montpellier, 1, 00133 Rome, Italy; paola.spitalieri@uniroma2.it (P.S.); federica.centofanti@gmail.com (F.C.); miky.murdi@hotmail.it (M.M.); scioli07@hotmail.it (M.G.S.); latini.andrea@hotmail.com (A.L.); gennaro.citro46@gmail.com (G.C.); orlandi@uniroma2.it (A.O.); botta@med.uniroma2.it (A.B.); novelli@med.uniroma2.it (G.N.); 2 Department of Systems Medicine, University of Rome Tor Vergata, Via Montpellier, 1, 00133 Rome, Italy; di.cesare@med.uniroma2.it; 3 Institute of Translational Pharmacology, CNR, 00133 Rome, Italy; antonio.rossi@ift.cnr.it; 4 The Donnelly Centre for Cellular and Biomolecular Research, University of Toronto, Toronto, ON M5S 3E1, Canada; shanemiersch@gmail.com (S.M.); sachdev.sidhu@gmail.com (S.S.S.); 5 Department of Molecular Genetics, University of Toronto, Toronto, ON M5S 3E1, Canada; 6 Renown Institute for Cancer, Nevada System of Higher Education, Reno, NV 89502, USA; pierpaolo.pandolfiderinaldis@renown.org; 7 IRCCS Neuromed, 86077 Pozzilli, Italy; 8 Department of Pharmacology, School of Medicine, University of Nevada, Reno, NV 89557, USA

**Keywords:** hiPSCs, hLORGs, SARS-CoV2 pseudovirus, neutralizing monoclonal antibody, synthetic peptide

## Abstract

The global health emergency for SARS-CoV-2 (COVID-19) created an urgent need to develop new treatments and therapeutic drugs. In this study, we tested, for the first time on human cells, a new tetravalent neutralizing antibody (15033-7) targeting Spike protein and a synthetic peptide homologous to dipeptidyl peptidase-4 (DPP4) receptor on host cells. Both could represent powerful immunotherapeutic candidates for COVID-19 treatment. The infection begins in the proximal airways, namely the alveolar type 2 (AT2) cells of the distal lung, which express both ACE2 and DPP4 receptors. Thus, to evaluate the efficacy of both approaches, we developed three-dimensional (3D) complex lung organoid structures (hLORGs) derived from human-induced pluripotent stem cells (iPSCs) and resembling the in vivo organ. Afterward, hLORGs were infected by different SARS-CoV-2 S pseudovirus variants and treated by the Ab15033-7 or DPP4 peptide. Using both approaches, we observed a significant reduction of viral entry and a modulation of the expression of genes implicated in innate immunity and inflammatory response. These data demonstrate the efficacy of such approaches in strongly reducing the infection efficiency in vitro and, importantly, provide proof-of-principle evidence that hiPSC-derived hLORGs represent an ideal in vitro system for testing both therapeutic and preventive modalities against COVID-19.

## 1. Introduction

Since its outbreak in 2019, Coronavirus Disease 2019 (COVID-19), caused by a novel severe acute respiratory syndrome coronavirus, SARS-CoV-2, has become a worldwide health emergency [1]. Due to the permissiveness of viral infection in numerous tissues, COVID-19 presents a wide set of symptoms, involving several human organ systems and leading to multiorgan failure and death [2]. As with other RNA viruses, SARS-CoV-2 is prone to genetic change for adapting to human hosts. Over time, mutations occur, resulting in the creation of many variants with distinct features that differ from the ancestral strain, called D614G. Since the original virus was reported, variants occurring in SARS-CoV-2 have been identified and characterized by increased transmissibility or virulence, decreased neutralization by antibodies obtained through natural infection or vaccination, the ability to escape detection, or a decrease in the efficacy of therapeutics and vaccines. The advent of these additional SARS-CoV-2 variations compromises the enormous progress accomplished in limiting the extent of this viral infection thus far, despite the exceptional speed with which vaccines against COVID-19 have been developed and the robust global mass immunization efforts that have been put into effect [3].

To date, synthetic monoclonal antibodies constitute efficacious immunotherapeutic candidates for COVID-19 treatment. They are built on a human IgG framework and bind to the SARS-CoV-2 Receptor Binding Domain (RBD) of Spike protein, thus competing for ACE2 binding and potently inhibiting virus entry into host cells [4,5,6]. Antibodies have a lengthy half-life in the blood and are usually free from side effects [6]. Thus, by adding two additional Fabs to the canonical IgG molecule, we have developed an innovative type of antibody, called tetravalent ultra-high affinity nAbs, exhibiting neutralization potencies higher than those of the bivalent IgG format [7].

Experimental data demonstrated that the tetravalent nAbs are more resistant to potential evading mutations, pointing out their potential as treatments against viruses such as SARS-CoV-2, which rapidly mutates under selective pressure [7].

On the other hand, peptide-based inhibitors are another promising treatment option for SARS-CoV-2, assuring high efficacy, specificity, and safety [8,9]. Peptides are able to simulate natural ligands. Because of their small size and specialized binding properties, they can physiologically disturb functional complexes. Based on the involvement of dipeptidyl peptidase-4 (DPP-4) in SARS-CoV-2 entry [10], a recent work tested the inhibitory effect of a synthetic peptide, designed on the DPP4 receptor sequence and used on Vero E6 cells in vitro model, in order to prevent SARS-CoV-2 from infecting host cells [11]. Computational investigations demonstrated that DPP4_270-295_ had a higher binding affinity for the RBD domain than the other synthetic DPP4-derived peptides, preventing the pseudovirus from intercepting the DPP4 receptor and other known receptors on target cells [11].

Clinical outcomes and studies using (2D) cultured cancer cell lines or ACE2 receptor-transgenic animal models for viral entry have led to our present understanding of the COVID-19 illness. Nevertheless, these models have limitations in replicating human physiology in order to comprehend fundamental molecular mechanisms governing host–pathogen interactions, viral replication kinetics, and tropism. As a result, robust and effective in vitro model systems for studying the pathophysiology of the SARS-CoV-2 infection are urgently needed.

As is known, SARS-CoV-2 infects the alveolar epithelial type 2 cells (AT2s), which are not easily accessible from patients. For this reason, the induced pluripotent stem cell (iPSCs)-derived AT2s represent a useful approach to simulate the initial apical infection process of the alveolar epithelium in vitro. Three-dimensional (3D) complex organoid structures, derived from hiPSCs, resemble the spatial organization and function of a lung-like tissue [12]. The organoids are multicellular formations that replicate the appropriate in vivo organ, recapitulating its key morphological and functional characteristics in vitro [13,14,15]. They better represent the physiological system than their 2D culture counterparts, as they show the signaling and the morphological cues that can occur within the human body [16]. Human lung organoids (hLORGs) include cell types and structures that imitate bronchi/bronchioles surrounded by lung mesenchyme and cells, expressing alveolar-cell markers used for SARS-CoV-2 infection [17]. In fact, due to their physiological expression of both angiotensin-converting enzyme 2 (ACE2) and dipeptidyl peptidase-4 (DPP-4) receptors, these cells are the best target for viral entry [18,19,20,21,22,23,24,25,26,27,28,29,30,31,32,33]. Moreover, hLORGs can be cultured in vitro for long periods (up to 85–100 days of cultures). Thus, these 3-dimensional human lung cell systems could correspond to a physiologically appropriate platform for SARS-CoV-2 infection modeling, which can help to speed up the discovery of an effective immunological intervention.

Here we tested, for the first time on human cells, two different strategies to inhibit virus entry, after the in vitro infection of hiPSCs-derived LORGs by SARS-CoV-2 S protein pseudovirus system carrying wild type (WT, B.1 lineage), D614G lineage, Alpha (B.1.1.7 lineage), Beta (B.1.351 lineage), Gamma (P.1 lineage), and Delta (B.1617.2 lineage) variants.

Specifically, we used a synthetic tetravalent antibody, Fab-IgG 15033-7, targeting the SARS-CoV-2 spike protein. In parallel, we utilized a synthetic peptide, DPP4_270–295_, based on recent evidence showing that DPP4 acts as a SARS-CoV-2 co-receptor and its interaction with the SARS-CoV-2 spike glycoprotein is a key factor for its virulence [10,11,29].

Finally, for both systems, we also evaluated the expression of interferons, cytokines, chemokines, and critical inflammasome pathway genes, induced by SARS-CoV-2 infection.

These strategies may represent an interesting therapeutic approach against SARS-CoV-2, ensuring high efficiency, specificity, and tolerability [8,9,11].

## 2. Materials and Methods

### 2.1. Human-Induced Pluripotent Stem Cell (hiPSC) Derivation and Maintenance

Human dermal fibroblasts were reprogrammed into human-induced pluripotent stem cells (hiPSCs), as previously reported [34,35,36,37]. A healthy donor subject (wild type, WT, 46 XX, age 46) has been recruited for dermal biopsy. Before participation, informed written consent was obtained. The project was approved by The Committees on Health Research Ethics of Tor Vergata Hospital (2932/2017) and in accordance with the Declaration of Helsinki.

hiPSC lines were manually picked, passaged on human embryonic stem cell-qualified Matrigel-coated plates (0.05 mg/mL; BD Biosciences, Franklin Lakes, NJ, USA), and cultured in mTeSR1 medium (Stem Cell Technologies, Vancouver, BC, Canada) with Y-27632 ROCK inhibitor (Stem Cell Technologies, Vancouver, Canada), and under a feeder-free condition, preserving the stability for over 30 and more passages. The stemness characteristics and karyotype were also verified (data not shown).

### 2.2. Differentiation into Human 3D Lung Organoids (hLORGs)

The direct differentiation process of hiPSC colonies to alveolar epithelial type II-like cells (AT2s) started when they were up to 70–80% confluent.

To generate lung progenitor cells, we used the STEMdiff™ Lung Progenitor Kit (Stemcell Technologies cat. # 100-0230, Vancouver, Canada), a serum-free medium system optimized for efficient and reproducible generation of lung progenitor cells through three stages of differentiation: (1) definitive endoderm, (2) anterior foregut endoderm, and (3) lung progenitor cells, according to the manufacturer’s instruction (see Figure S1A). Briefly, hiPSC were first seeded in mTeSR™1 (Stemcell Technologies, Vancouver, Canada). On Day 1, the differentiation was initiated with Medium DE-1 (STEMdiff™ Endoderm Basal Medium, containing Supplement MR and Supplement CJ). Subsequently, on days 2 and 3, the medium was changed to Medium DE-2 (STEMdiff™ Endoderm Basal Medium, containing Supplement CJ) for definitive endoderm patterning. On Day 4, to initiate anterior foregut endoderm patterning, the endoderm monolayer was disaggregated for 1–2 min at room temperature with GCDR and passaged at a ratio between 1:2 to 1:6 into 6 well plates pre-coated with hES-qualified Matrigel (Corning, New York, NY, USA) and cultured in Medium LP-1 (STEMdiff™ Lung Basal Medium, Lung Supplement (10X), and Supplement 1 and Y-27832. Finally, at Day 7, the cells were differentiated into the lung progenitor stage with Medium LP-2 (STEMdiff™ Lung Basal Medium, Lung Supplement (10×), and Supplement 2. On day 15 of differentiation, lung progenitors were disaggregated with 0.05% trypsin–EDTA and replated at a density of ~125–200 cells/μL in 3D in undiluted growth factor-reduced Matrigel (Corning, New York, NY, USA). Distal/alveolar differentiation of cells was performed in “CK+DCI” medium (alveolosphere medium), consisting of 3 μM CHIR99021 (Sigma-Aldrich, San Louis, Missouri, MO, USA), 10 ng/mL rhKGF, 50 nM dexamethasone (Sigma-Aldrich, San Louis, Missouri, MO, USA), 0.1 mM 8-Br-cAMP (Sigma-Aldrich, San Louis, Missouri, MO, USA), and 0.1 mM 3-Isobutyl-1-methylxanthine (IBMX;Sigma-Aldrich, San Louis, Missouri, MO, USA) in cSFDM (complete Serum-Free Differentiation Medium: Iscove’s Modified Dulbecco’s Medium (IMDM), Ham’s F12, GlutaMAX, B27 supplement, 7.5% BSA, N2 supplement, 50 μg/mL ascorbic acid, diluted MTG (final concentration, 4.5 × 10^−4^ M), and Anti-Anti (final concentration, 200 ng/mL), with a brief period of CHIR99021 withdrawal on days 31–35 to achieve alveolar maturation, as previously described [38]. The resulting alveolospheres were further matured and maintained in culture for up to 105 days by successive passages in Matrigel GFR (Corning, New York, NY, USA) droplets at a density of 400–800 cells/μL, refeeding every other day in the CK+DCI medium. For the maintenance of longer culture, the organoids were re-embedding every 2 weeks in Matrigel GFR. We utilized 60 days until 105 old lung organoids to characterize for the expression of specific markers and SARS-CoV-2 infection studies (see Figure S1).

### 2.3. hLORG Characterization

For confocal microscopy, hLORGs in solution (PBS-glycerol, 1:1) were dropped into specific imaging 8 multi-well chambers with a coverslip glass bottom (Ibidi) and analyzed by using a FV 1000 Olympus confocal microscope. Imaris software (version 9.8; Bitplane, Switzerland) was used for 3D rendering.

Immunofluorescence analyses were performed on hLORG after Matrigel removal by Dispase. Dissociated organoids in Dispase were transferred to a 15-mL conical tube, with 10 mL of DMEM/F12 added and washed two times, centrifuged at 300 g for 4 min at 4 °C, and the supernatant was aspirated and fixed for 15 min in 4% paraformaldehyde. Then, samples were embedded in OCT and sectioned between 5 and 8 µm on silanized glass slides. After permeabilization (10 min in 0.2% Triton X-100), samples were blocked for 30 min in 1% BSA and incubated with primary antibodies 1h at room temperature (mouse anti-tubulin 1:1000 and rabbit anti-actin 1:200, Merk, Darmstadt, Germany; mouse anti-DPP4 1:50, BioLegend, San Diego, California, CA, USA). After washing with PBS, the samples were incubated with specific secondary antibodies (Nordic Immunology, Susteren, Netherland) in the presence of Hoechst (1:1500, Nordic Immunology) for 30 min at room temperature. The slices were analyzed under a fluorescence microscope (Eclipse E600; Nikon, Tokyo, Japan) and images were acquired by using a digital camera and ACT-1 software (Nikon, Tokyo, Japan).

For histological analysis, the organoids were fixed in 4% formalin followed by dehydration, paraffin embedding, sectioning, and standard H&E staining. The immunohistochemistry was performed using goat anti-ACE2 antibody (1:60, AF933, R&D systems, Minneapolis, Minnesota, MN, USA), and rabbit anti-SFTPC (1:1000, ab3786, Chemicon, Temecula, California, CA, USA). After the incubation with specific biotin-conjugated secondary antibodies and streptavidin-HRP (Nordic), the immunoreaction was revealed by using the chromogen 3,3′-diaminobenzidine (DAB). The images were captured on the Eclipse E600 microscope.

To perform the Flow Cytometry Analysis, dissociated hLORG were stained for cell surface expression of CD26 (Biolegend, # 302706, Clone BA5b, PE anti-human CD26 antibody) to standard procedures. Intracellular staining was performed according to the manufacturer’s instructions with an intracellular fixation and Permeabilization Buffer Set (eBioscience, Invitrogen, Waltham, Massachusetts, MA, USA). Briefly, dissociated hLORG were fixed and after permeabilization, they were stained with rabbit anti-human prosurfactant protein C polyclonal antibody (proSP-C, #AB3786, Chemicon International), followed by goat anti-rabbit IgG FITC conjugated (Thermoscientific, Waltham, Massachusetts, MA, USA #31583). The gating was based on isotype-stained controls. Samples were acquired with a FACSCanto II (Becton Dickinson, Franklin Lake, New Jersey, NJ, USA) and analyzed with FlowJo software (TreeStar Inc., Ashland, OR, USA). The FACS plots shown represent single-cells based on forward-scatter/side-scatter gating.

For transmission electron microscopy (TEM), hLORG were fixed in Karnovsky’s solution and processed and embedded in EPON 812. Ultrathin sections were counterstained with uranyl acetate and lead citrate, and photographed with a H-7100FA Hitachi transmission electron microscope (Japan).

### 2.4. Pseudotypes SARS-2-S Infection and Treatment with Monoclonal Antibody Fab-IgG 15033-7 and DPP4 Peptide

For viral infection, the hLORGs were treated enzymatically, released from Matrigel, followed by gently pipetting alveolospheres in order to “open” them to allow viral access to the apical membrane. Successively, they were infected with 50 μL of VSVpp.SARS-2-S virus or 25 μL of Pseudotype Lentivirus SARS-2-S (BPS Bioscience #78215) and incubated at 37 °C and 5% CO_2_ for 2 h [11]. Then, the alveolospheres were plated in Matrigel GFR 3D droplets at a cell density of ~1200–1600 cells/μL using CK+DCI media with Y-27832 for the first 48 h and then analyzed.

For treatments with the monoclonal antibody Fab-IgG 15033-7 and DPP4 peptide, the VSVpp.SARS-2-S and Pseudotype Lentivirus SARS-2-S viruses are first incubated with Fab-IgG 15033-7 (200 ng/mL) or DPP4 peptide (200 μg) for at least 30 min at 37 °C [7,11] and then are added to the organoids (previously disrupted into small clumps) and left to act for 2 h at 37 °C. The hLORG are then incorporated in drops of Matrigel GFR at a cell density of ~1200–1600 cells/μL, using CK+DCI media with Y-27832, which are left to grow for the first 48h and then analyzed. The infections with the SARS-CoV-2 pseudovirus containing eGFP (with and without Fab-IgG 15033-7 and DPP4 peptide) were evaluated at 48 h pi, both taking bright and fluorescent field images of hLORG and for testing luciferase activity, according to the manufacturer’s instructions. Conversely, the infections with the Pseudotype Lentivirus SARS-2-S (with and without Fab-IgG 15033-7 and DPP4 peptide) were evaluated at 48 hpi for luciferase activity only, according to the manufacturer’s instructions. The activity of firefly luciferase was quantitatively assessed, using the Promega Luciferase Assay System (Promega, Madison, Wisconsin, WI, USA, E1501), and the luminescence was evaluated using a luminometer, following the manufacturer’s instructions.

### 2.5. Gene Expression Analysis

Trizol Reagent (Invitrogen Life Technologies Corporation, Carlsbad, CA, USA) was used to extract total RNA from cells, according to the manufacturer’s instructions. Total RNA samples were treated with DNase I-RNase-free (Ambion, Life Technologies Corporation, Foster City, CA, USA) to remove genomic DNA contamination. One µg of RNA was reverse transcribed and used in RT-qPCR using the Life Technologies Corporation’s High-Capacity cDNA Archive kit (Foster City, CA, USA). SYBR Green was used to assay mRNAs (Life Technologies Corporation, Foster City, CA, USA). As reference genes, 5.8S and GAPDH were employed. Primer sequences will be given upon request. The 2^−(ΔCt)^ and comparative ΔΔCt methods were used to quantify relative gene expression levels.

### 2.6. Preparation of Pseudotype Particles, Monoclonal Antibody Fab-IgG 15033-7, and Synthetic Peptide DPP4_270–295_

The SARS-CoV-2 S protein pseudovirus system (kindly provided by Hoffmann lab, German Primate Center–Leibniz Institute for Primate Research, Gottingen, Germany) has been used for mimicking SARS-CoV-2 infection. The Vesicular Stomatitis Virus (VSV) pseudovirus system was employed to produce the SARS-CoV-2 pseudovirus, which displays the SARS-CoV-2 spike protein (S) on the VSV particle surface. S protein, in fact, represents the surface protein responsible for the virus attachment and entry into the target cells by the fusion of the viral membrane with cellular membranes [23,39].

We used a replication-deficient VSV vector that lacks the genetic information for VSV-G (VSV*∆G-fLuc) (kindly donated by the Hoffmann Lab, German Primate Center—Leibniz Institute for Primate Research, Gottingen, Germany) to generate SARS-CoV-2 S-pseudotyped particles. The infectious titers of VSV*∆G-fLuc were estimated as fluorescence-forming units per milliliter (ffu/mL) on mefepristone-induced BHK-G43 cells. SARS-2-S plasmids expressing the WT (B.1 lineage) protein as well as the variants, Alpha (B.1.1.7 lineage), Beta (B.1.351 lineage), and Gamma (P.1 lineage) SARS-CoV-2 spikes (a kind gift from Hoffmann Lab, German Primate Center–Leibniz Institute for Primate Research, Gottingen, Germany), were transfected into 293T cells (CRL1573). For the Delta variant (B.1.617.2; Genbank Accession #QHD43416.1 with B.1.617.2 mutations), we used a pseudotyping system based on Lentiviruses (BPS Bioscience, San Diego, California, CA, USA).

The infectious titers of VSV*DG-fLuc particles pseudotyped with SARS-CoV2 S proteins were calculated by counting the GFP positive cells after the infection of 293T-ACE2 cells with serial diluted virus supernatant. Titers were expressed as fluorescence-forming units per milliliter (ffu/mL) and infections were performed by using 250 ffu/well in 50 µL.

After 24 h from transfection, cells were infected with VSV*DG-fLuc (m.o.i. of 3 ffu/cell). Virus inocula were removed after 1 h of incubation at 37 °C and monolayers were washed twice with DMEM. Cell were then incubated with DMEM containing 5% FCS and treated with anti-VSV-G antibodies (I1, mouse hybridoma supernatant from CRL-2700) to neutralize leftover input virus. At 24 h post infection, the pseudotyped particles were extracted, centrifuged to remove cellular debris, and kept at −80 °C until use.

The tetravalent Ab 15033-7 was produced and purified as described [7], while DPP4_270–295_ has been already tested in our lab, as reported [11].

### 2.7. Statistical Analyses

Molecular experiments and luciferase assay were performed in technical triplicates and data were analyzed using GraphPad Prism 8 and SPSS version 19 (IBM Corp, USA). The difference between groups was tested by a paired Student *t*-test and one-way ANOVA test. Values displayed in the figures represent the means of three independent experiments ± standard deviation (SD). Statistical significance was established at * *p* < 0.05, ** *p* < 0.01, and *** *p* < 0.001.

## 3. Results

### 3.1. Production, Molecular Characterization, and Morphological Analysis of hiPSC-Derived hLORGs

**Figure 1:**
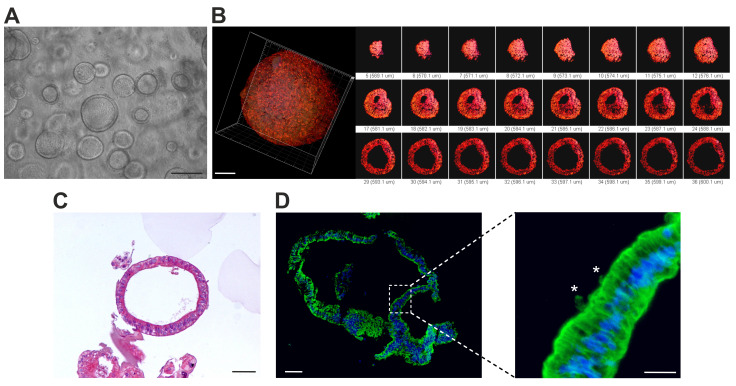
**In vitro model of human lung organoids (hLORGs) derived by hiPSCs**. (**A**) Phase–contrast microscopy of alveolospheres embedded in 3D Matrigel at day 60 of culture. Scale bar 200 µm. (**B**) Confocal images confirm the spheroidal structure of an hLORG (at day 105) showing a peculiar well-developed inner cavity: in red staining of cytoskeleton by total actin. Scale bar 60× and 30 µm, respectively. (**C**) Haematoxylin and eosin-stained hLORG cross-section showing the typical epithelial morphology. Scale bar 50 µm. (**D**) Immunofluorescence images show the overall α-tubulin distribution (green) of an hLORG displaying a prominent epithelial structure (nuclei, blue). At higher magnification, it is visible in the presence of ciliated cells (*). Scale bar 50 µm and 25 µm, respectively.

To create a human 3D model system that could mimic SARS-CoV-2 infection in vitro, we used a direct differentiation process to convert hiPSCs to lung organoids (hLORGs). This process was designed to mimic the sequence of developmental milestones that occur during in vivo human fetal organogenesis, from early endodermal progenitors to increasingly mature stages of alveolar development. Once obtained, organoid models were genetically stable (data not shown) and have been expanded over long periods of time, up to 105 days, providing an excellent model for the study of SARS-CoV-2 infection. We created self-renewing epithelial sphere cultures in 3D Matrigel, which are made of organ-specific cell types that self-organize through spatially constrained lineage commitment [13,40]. 3D hLORGs were composed of a variety of cell types and compartments resembling a fetal lung [41,42]. hLORGs exhibited a spherical shape with a typical diameter of 200–300 μm (Figure 1A).

A confocal 3D reconstruction, after cytoskeleton immunostaining with actin antibody, confirmed the spheroidal spatial distribution of cells constituting hLORGs and showed a peculiar well-developed inner cavity, like pulmonary alveoli (Figure 1B). Hematoxylin and eosin (H&E) staining revealed a prominent epithelial structure in hLORGs by day 60 (Figure 1C). Immunostaining for α-tubulin confirmed the characteristic epithelial morphology with the presence of ciliated cells (Figure 1D).

**Figure 2:**
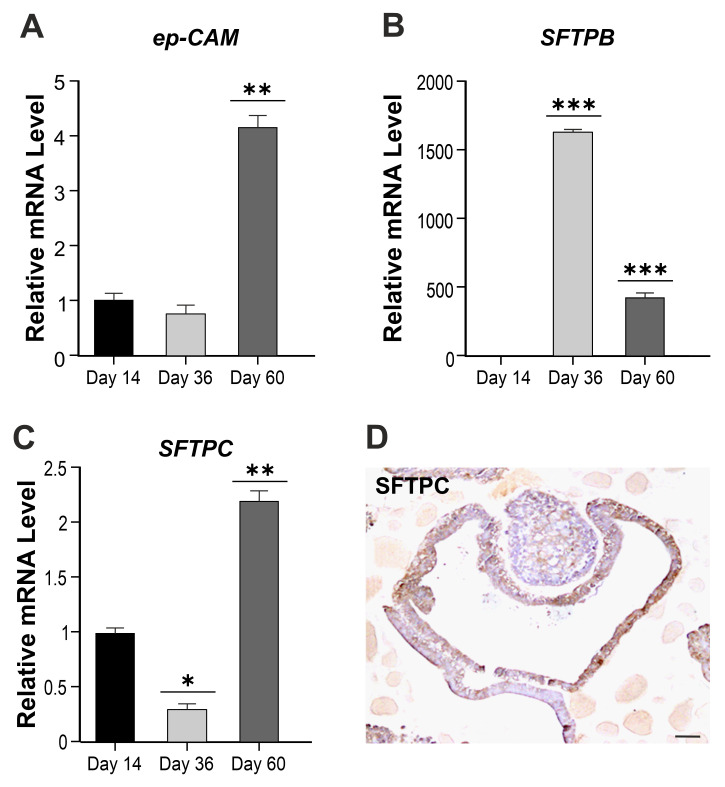
**Characterization of hLORGs**. (**A**–**C**) RT–qPCR analyses of lung epithelial cell marker-*EPCAM* and *AT2* cell genes *SFTPB*, *SFTPC* in hLORGs at days 14, 36, and 60 of differentiation. Data are representative of three independent experiments and reported as mean ± SD. * *p* < 0.05, ** *p* < 0.01, and *** *p* < 0.001 by Student’s *t*-test. (**D**) Immunohistochemistry analysis of SFTPC protein in hLORGs at day 60 of differentiation. Scale bar 50 µm.

Quantitative RT-qPCR gene expression analyses from day 14 until day 60 of the hLORG differentiation also demonstrated the presence of transcripts encoding for lung epithelial cell adhesion molecules (*EPCAM*) and surfactant proteins B and C (*SFTPB* and *SFTPC*), two canonical alveolar type 2 pneumocyte markers. These genes are mainly expressed in alveolar type 2 and non-ciliated bronchiolar cells and are also essential for lung function, reducing the surface tension of fluids that coat the lung. Specifically, we found a significant increase in *EPCAM* expression after 60 days of differentiation, as compared to hLORG precursors at day 14 (** *p* < 0.01) (Figure 2A).

Regarding alveolar type 2 markers, we observed an opposite correlation in the expression of *SFTPB* and *C* along days. In fact, after 36 days of differentiation, the SFTPB marker increased (*** *p* < 0.001) (Figure 2B), while *SFTPC* decreased with respect to day 14 (* *p* < 0.05) (Figure 2C). The trend reversed at day 60, as expected, and the differential expression results to be statistically significant (*** *p* < 0.01; ** *p* < 0.001) (Figure 2B,C). An immunohistochemistry analysis of SFTPC protein has also been performed to localize this protein within hLORG (Figure 2D). The same analysis, reported as absolute value, is shown in Figure S2, evidencing a *SFTPB/C* ratio equal to 16:1.

**Figure 3:**
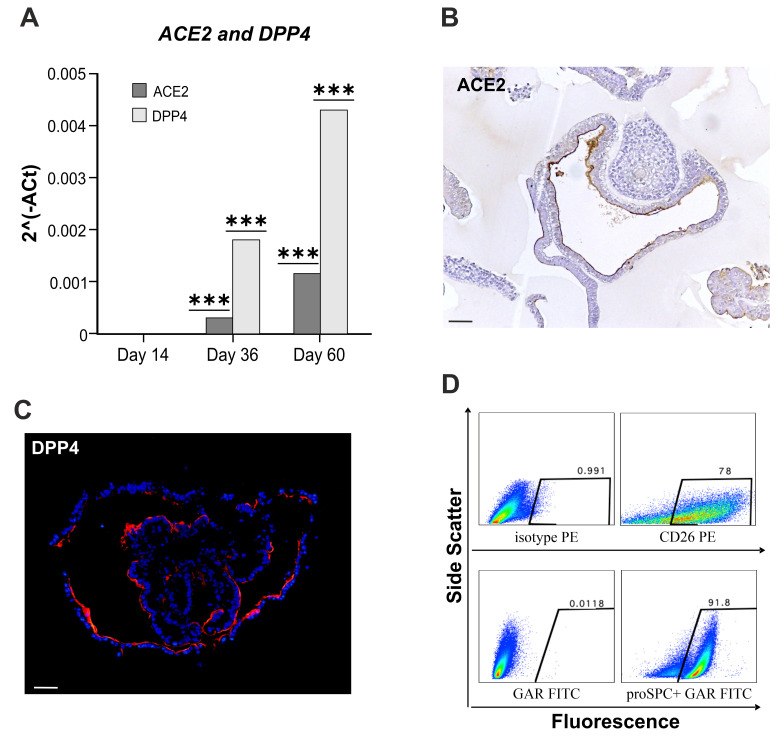
**Expression of spike receptors**. (**A**) RT–qPCR analyses of SARS-COV2 receptors (i.e., *ACE2*, *DPP4*) in hLORGs at days 14, 36, and 60 of differentiation. Data are representative of three independent experiments and reported as mean ± SD. *** *p* < 0.001 by one-way ANOVA test. (**B**,**C**) ACE2 and DPP4 protein expression in hLORGs at day 60 of differentiation by immunohistochemistry/immunofluorescence. Scale bar 50 µm. (**D**) FACS analysis of surface DPP4 (CD26) and intracellular SFTPC (proSPC) in hLORG cells. In the first column the control plots (mouse IgG2a isotype PE and goat anti Rabbit FITC) are depicted, in the second column the frequencies of DPP4 positive cells (CD26) (**upper row**) and of SFTPC (proSPC) (**lower row**) are reported.

The ACE2 and DPP4 receptors expression were then evaluated. Both transcripts resulted to be increased in hLORGs at days 36 and 60 of differentiation (*** *p* < 0.001). Interestingly, a boost in the expression of *DPP4* was detected at 60 days (Figure 3A).

This result is surprising, as the ACE2 receptor is universally recognized to be the main receptor of the SARS-COV-2 spike glycoprotein, and the expression of DPP4 in hiPSC-derived hLORGs has never been described so far [16,43,44]. Importantly, the ACE2 receptor, typically expressed at the apical surface of AT2 cells [20], was clearly detected in our differentiated hLORGs, as visualized by immunostaining (Figure 3B) [45]. The immunofluorescence analysis of DPP4 confirmed its strong positivity along the apical surface of the alveolar cells (Figure 3C), as described in the literature [43].

DPP4 (CD26) and SFTPC levels were lastly quantified by flow cytometry, which demonstrated that 78% and 92% of cells expressed these proteins, respectively (Figure 3D).

Moreover, the expression of the genes encoding for proteins known to influence SARS-CoV-2 entry (*ACE2*, *DPP4*, *CD147*, *PCSK3*, and *TMPRSS2*) was evaluated by real time RT-PCR and shown in Figure S3. As shown, except for CD147, also known as basigin and considered a new receptor facilitating SARS-CoV infection, all other markers are much less expressed than in adult tissue, confirming the immaturity of the iPSCs-derived hLORGs.

All the above data confirmed the presence of AT2 cells expressing specific SARS-CoV-2 receptors within our hLORGs.

### 3.2. hLORGs are Permissive to SARS-CoV-2 Infection

**Figure 4:**
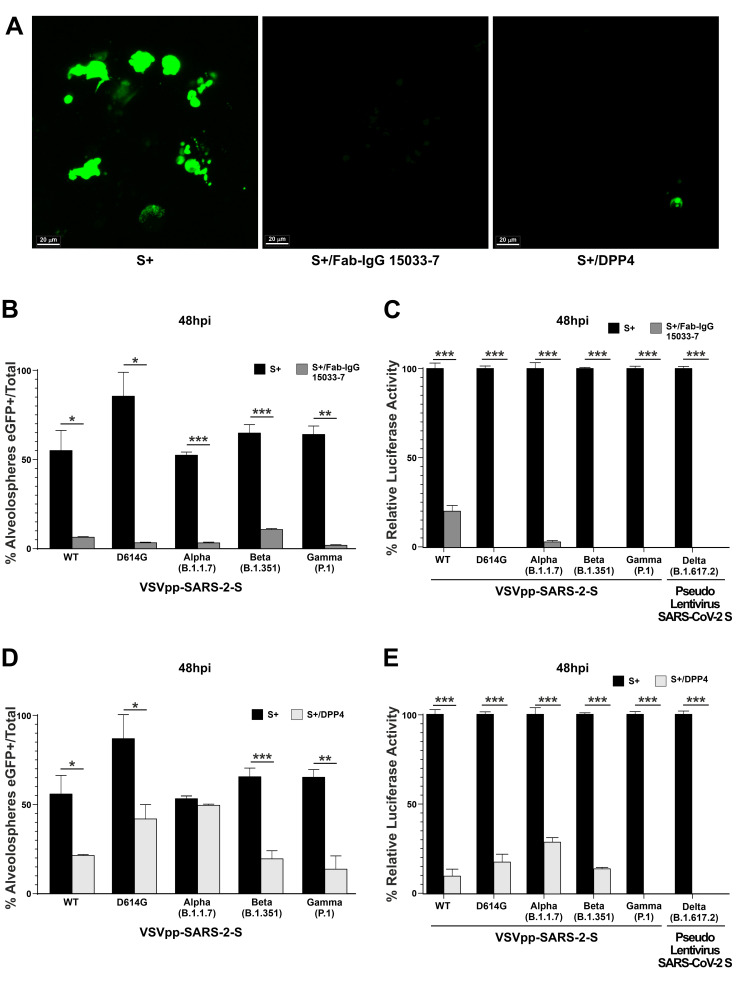
**Fab-IgG 15033-7 and DPP4_270-295_ synthetic peptide are able to inhibit pseudotype SARS-2-S virus infection**. (**A**) eGFP expression detected by confocal image representative of one alveolosphere infected by VSVpp.SARS-2-S virus (S+). When alveolospheres were treated with antibody neutralizing Fab-IgG 15033-7, eGFP expression is not observed, while when treated with peptide DPP4_270–295_, the eGFP expression is much lower than that shown with pseudoviruses alone. Scale bar 20 µm. (**B**) Percentage of eGFP positive alveolospheres, 48 h post infection (hpi) with VSVpp.SARS-2-S WT protein and D614G, Alpha (B.1.1.7 lineage), Beta (B.1.351 lineage), Gamma (P.1 lineage) variants and treated with neutralizing antibody Fab-IgG 15033-7. *n* = 25–30 fields from three biological replicates and two different observers, * *p* < 0.05, ** *p* < 0.01, and *** *p* < 0.001 by one-way ANOVA test. (**C**) Transduction efficiency was quantified by measuring virus-encoded luciferase activity in cell infected for 48 h with pseudotypes SARS-2-S WT or variants alone and in combination with Fab-IgG 15033-7. Data are expressed as the percentage of infection and the average data from three biological replicates is presented. Error bars indicate the standard deviation (±SD). *** *p* < 0.001 by one-way ANOVA test. (**D**) Percentage of eGFP positive alveospheres 48 hpi with VSVpp.SARS-2-S WT protein and its variants and treated with synthetic peptide DPP4_270–295_. Two different observers analyzed 25-30 fields from three biological replicates; * *p* < 0.05 and ** *p* < 0.01 by one-way ANOVA test. (**E**) Transduction efficiency was quantified by measuring virus-encoded luciferase activity in cell infected for 48 h with pseudotype SARS-2-S WT or variants alone and in combination with synthetic peptide DPP4_270–295_. Data are expressed as percentage of infection and the average data from three biological replicates is presented. Error bars indicate the standard deviation (±SD). *** *p* < 0.001 by one-way ANOVA test.

Three-dimensional alveolospheres were treated to permit the virus to enter through the apical membrane (see Section 2). Then, alveolar cultures were infected by the pseudovirus SARS-2 (S+) or pseudovirus SARS-2 S without the spike protein (S−, as controls) and the cells were harvested for analysis. eGFP was detected as early as 48 h post-infection (hpi) in the virus-exposed (Figure 4A), but not in control alveolospheres (S−) (data not shown).

In addition, microscopic analysis of haematoxylin and eosin-stained sections of infected alveolopheres revealed cell damage features compared with not infected ones (Figure S4). In particular, an alveolocyte hyperplasia with enlarged nuclei and prominent nucleoli with hyaline deposition, suggesting a fibrotic process, was observed (see Figure S4). Electron microscopy images further revealed the presence of the lamellar bodies, a characteristic of relatively mature type 2 pneumocytes, and some viral particles within a single membrane vesicle (Figure S5).

### 3.3. A Potent Neutralizing Antibody is Effective in Preventing SARS-CoV-2 Pseudovirus Infection in hLORGs

The effectiveness of the Fab-IgG 15033-7 SARS-CoV-2 neutralizing antibody, which is engineered to bind directly to the viral spike glycoprotein [7], was then investigated. As recently reported, Fab-IgG 15033-7 inhibits ACE2 from binding to the RBD by direct steric hindrance, and the simultaneous binding of both IgG arms to the S-protein trimer likely boosts potency via avidity effects [7,46]. The antibody was incubated with the SARS-2-S pseudotype WT and the variants described above, and after 30 min the mixture was used to infect hLORGs.

The infection efficacy, as well as the neutralizing capacity of the monoclonal antibody, was quantified by assessing both eGFP signal and luciferase assay (Figure 4B,C). Specifically, Fab-IgG 15033-7 exhibited high neutralizing capacity, as shown in Figure 4B, in which the percentage of alveolospheres positive for eGFP signal was reported, comparing the S+ infection with and without the monoclonal antibody.

The efficiency of the VSVpp.SARS-2-S WT viral infection was 55%; this value decreased to 6.5% in cells treated with Fab-IgG 15033-7. The efficiencies of infection obtained using the pseudotype variants with or without Fab-IgG 15033-7 were as follows: 86% for the D614G variant versus 3.2% in cells previously treated with Fab-IgG 15033-7, 53.4% for the Alpha variant versus 3.7% with Fab-IgG 15033-7; 65% for the Beta variant versus 11% with Fab-IgG 15033-7, and 49% for the Gamma variant versus 1.8% with Fab-IgG 15033-7 (Figure 4B).

In parallel, the neutralizing capacity of Fab-IgG 15033-7 was also evaluated by a luciferase assay, which quantifies the capacity of virus cell entry. As observed in Figure 4C, the capacity of pseudovirus WT and its variants (i.e., D614G, Alpha, Beta, Gamma, Delta) to enter into target cells was strongly inhibited. Specifically, the infection rate of cells treated with Fab-IgG 15033-7 was: 20% for WT, 3.5% for Alpha variant, 0.19% for Delta, and 0% in the remaining pseudotype lineages tested (*** *p* < 0.001) (Figure 4C).

### 3.4. A Synthetic Peptide Effectively Inhibits Virus Entry in hLORGs

In addition, the synthetic DPP4_270–295_ peptide was employed to evaluate its ability to inhibit virus entry into hLORGs. Our results demonstrated a variable capacity of DPP4_270–295_ for inhibiting virus infection (Figure 4D). In fact, the peptide DPP4_270–295_ reduced the ability of the virus to enter into AT2 cells from 55% to 21% after infection with WT pseudotype and from 86% to 47% after infection with D614G, from 54% to 49% after infection with Alpha, from 65% to 23% after infection with Beta, and from 49% to 14% after infection with Gamma (Figure 4D). The luciferase assay confirmed a similar trend and specifically a percentage of infection reduced to 10% (WT), 17% (D614G), 29% (Alpha), 15% for Beta, 0% for Gamma, and 2% for the Delta variant (*** *p* < 0.001) (Figure 4E).

Overall, these data confirmed the capacity of both systems to interfere with virus entry into host cells, even if with different efficacy.

### 3.5. Expression Profiles of Genes Involved in Innate Immunity and Inflammation

Lastly, we also compared the expression in hLORGs of some immunity-related genes 48 h after VSVpp.SARS-2-S infection, with (S+) and without (S−) the spike protein of SARS-CoV-2.

**Figure 5:**
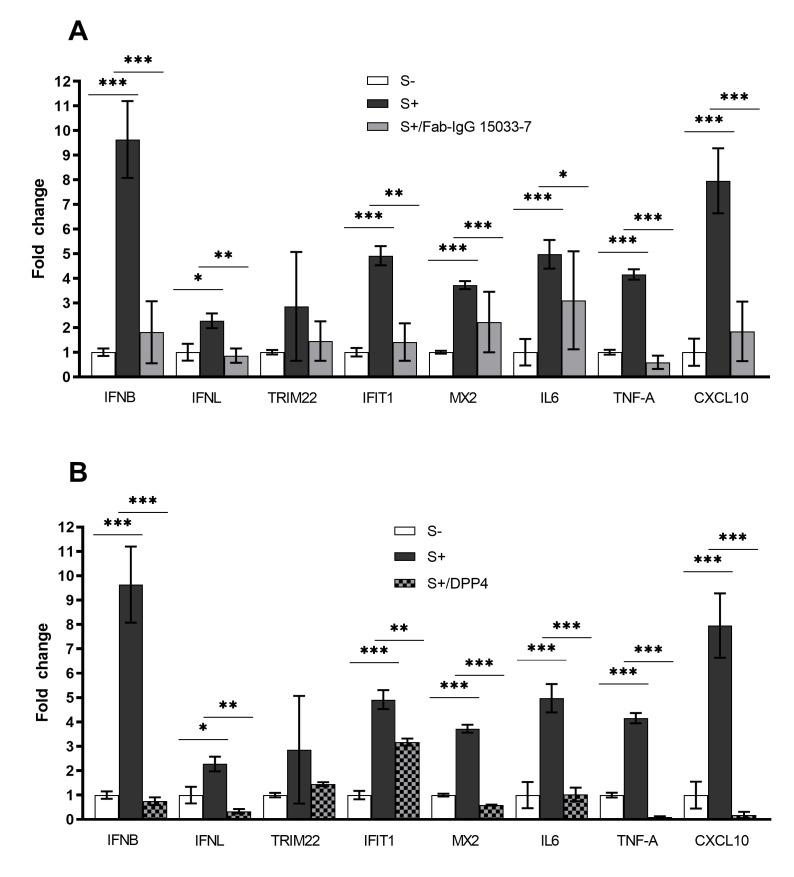
**Expression of immunity-related genes in hLORGs**. (**A**) Expression of immunity-related genes in hLORGs 48 hpi, with (S+) and without (S−) spike protein of VSVpp.SARS-2-S, and following treatment with Fab-IgG 15033-7. Bar graph shows expression of immune response genes and cytokines, quantified by qRT-PCR at 48 hpi.* *p* < 0.05, ** *p* < 0.01, and *** *p* < 0.001 by one-way ANOVA test. (**B**) Expression of immunity-related genes in hLORGs 48 h after pseudovirus infection, with (S+) and without (S−) spike protein of SARS-CoV-2, and following treatment with DPP4_270–295_ peptide. Immune response genes and cytokines expression of was quantified by qRT-PCR at 48 hpi. Data are from three independent experiments and represented as mean± SD. * *p* < 0.05, ** *p* < 0.01, and *** *p* < 0.001 by one-way ANOVA test. These data showed that the anti-inflammatory capacity of both treatments (Fab-IgG 15033-7 and DPP4_270–295_ peptide) was useful to ameliorate the clinical response to SARS-CoV-2.

We confirmed, by RT-qPCR, that pseudo-SARS-CoV-2 induced type I *(IFNβ)* and III *(IFNλ1)* IFNs expression, the first protection against viral infections, as well as the expression of interferon-stimulated genes (*IFIT1, TRIM22, and MX2*), which counteract viral replication, transcription, and translation in infected and uninfected cells and stimulate the adaptive immune response. The overexpression was strongly significant for *IFNβ, IFIT1*, and *MX2* (*** *p* < 0.001), while the increase of *TRIM22* expression level did not reach statistical significance (Figure 5). In addition, we observed that mRNAs levels of pro-inflammatory chemokines and cytokines (*CXCL10, IL-6,* and *TNF-α*) were significantly upregulated in pseudo-SARS-CoV-2-infected hLORGs (*** *p* < 0.001) (Figure 5).

Lastly, to explore the effect of Fab IgG 15033-7 on the innate immune response and support its efficacy against VSVpp.SARS-2-S, we evaluated the expression of immunity-related genes in infected hLORGs after the Fab-IgG 15033-7 treatment. We observed a significant decrease in mRNA levels of *type I* (*** *p* < 0.001) and *type III IFNs* (** *p* < 0.01), compared to those of infected cells not treated with Fab IgG 15033-7 (Figure 5A). In particular, Fab-IgG 15033-7 seemed to induce a five-fold and three-fold decrease in *IFNβ* and *IFNλ1* expression, respectively. As expected, the hLORGs treated with Fab-IgG 15033-7 also demonstrated a significant downregulation of interferon-stimulated genes (*IFIT1* and *MX2*) and of pro-inflammatory chemokines and cytokines (*CXCL10, IL-6,* and *TNF-α*). Indeed, the decrease of IFNs induces a lower immune response. This downregulation is more evident in the levels of *TNF-α* and *CXCL10* mRNAs that are seven-fold and four-fold lower, respectively, compared to those of infected cells not treated with Fab IgG 15033-7 (Figure 5A). The same analyses were performed in infected hLORGs previously treated with the DPP4_270–295_ synthetic peptide. The results always demonstrated a significant decrease in the *IFNβ* (*** *p* < 0.001) and *IFNλ1* expression levels (** *p* < 0.01) in DPP4_270–295_ treated cells (Figure 5B). Furthermore, the expression of interferon-stimulated genes and of genes encoding pro-inflammatory chemokines and cytokines also appear to decrease following treatment with DPP4_270–295_ peptide. In particular, we observed that the expression levels of *TNF-α* and *CXCL10* in cells treated with DPP4_270-295_ peptide were more than 40-fold lower than in VSVpp.SARS-2-S infected cells (*** *p* < 0.001) (Figure 5B).

## 4. Discussion

SARS-CoV-2 infection mainly involves the respiratory system spreading from one person to another by droplets of saliva. In 87% of cases, the lung tissue collected during the autopsy of patients who tested positive for COVID-19 showed typical diffuse alveolar damage features. In addition, type II pneumocyte hyperplasia, airway inflammation, and hyaline membranes were often observed in alveolar zones. The virus was found in airway epithelium and type 2 pneumocytes, which are rich in angiotensin-converting enzyme 2 (ACE2) and DPP4 receptors [47,48].

Human lung organoids (hLORGs) are useful as in vitro models for the study and for potential therapeutic treatments of SARS-CoV-2. They are produced from hiPSCs and are a one-of-a-kind model of AT2 cells in 3D complex organoid structures, containing various cell types. These cells’ assembly with spatial organization and function is similar to that of fetal lung tissue.

Several researches utilizing stem cell-derived organoids have supplied valuable insight into the SARS-CoV-2 infection of diverse cell types and host responses, as well as aiding in the development of therapeutic candidates [16,21,24,31,42,49,50,51,52]. In vitro cell models have been used for evaluating aspects of viral entrance, life cycle, tropism, and pathogenesis of the COVID-19 infection. The human cell lines HEK293T, Calu-3, Caco-2, and Huh7, for example, are permissive for viral infection in vitro [53], but they do not accurately mirror human physiological and pathological responses [54]. Human primary cells [22] and adult organoids [31,33] may better model SARS-CoV-2 infection, although they are limited by the scale that is required for high-throughput drug screening.

In this work, we obtained three-dimensional hLORG models including AT2-like cells that express ACE2 and DPP4, as well as the presence of the surfactant proteins SPC and SPB. ACE2 is mostly expressed by AT2 cells, according to recent single-cell transcriptome analyses [22,55]. Importantly, we demonstrated for the first time the expression of the DPP4 receptor, a type II transmembrane glycoprotein. Recent studies have discovered a link between dipeptidyl peptidase-4 (DPP4/CD26) and ACE2, implying that both membrane proteins could be involved in virus entrance pathogenicity [55]. DPP4 is mainly expressed in alveolar epithelial type I and II cells, as well as alveolar macrophages. The expression of DPP4 and SPC proteins, as well as the presence of microvilli, but most importantly the presence of lamellar bodies inside the hLORGs, suggest that we have obtained an in vitro model of relatively mature AT2-like cells, useful as infection targets in our investigations [56,57,58].

Regarding the role played by DPP4 with respect to virus infection, literature data are quite conflicting. On one side some studies highlight the finding that the S1 domain of the COVID-19 spike glycoprotein potentially interacts with the human CD26 [59], indicating DPP4 as one of the functional receptor of the human coronavirus [60,61,62]. Studies based on docking complex model of the SARS-CoV-2 spike glycoprotein and DPP4 showed a large interface between the proteins, suggesting additional virus–host interaction on the cell surface besides ACE2, which remains the main access door used by SARS-CoV-2 [63,64].

Nevertheless, the question on whether DPP4 is directly involved in the SARS-CoV-2 cell adhesion/virulence remains open, and although a direct involvement of DPP4 in SARS-CoV-2 infection needs to be clarified, there is also evidence suggesting that DPP4 inhibitors modulate inflammation and exert anti-fibrotic activity due to SARS-CoV-2 infection [65]. It was therefore hypothesized that DPP4 could act as a co-receptor. In fact, it is known that the viruses to enter into hosts require multiple transmembrane proteins apart from the primary receptor [44,59,61,66,67]. On the other side, there are conflicting studies assessing that the RBD of the S1 domain did not absolutely exhibit binding activity to DPP4 in human cells. Researchers have published data that did not support DPP4 being a significant receptor for SARS-CoV-2. In fact, even if the overall binding mode of the RBD of SARS-CoV-2 to DPP4 is predicted to be similar to that observed in the MERS-CoV-DPP4 complex, important differences in the amino acid sequences of SARS-CoV-2 and MERS-CoV result in substantially weakened interactions with DPP4. Moreover, the fact that cells expressing the ACE2 receptor can be infected by SARS-CoV-2, but not if they express DPP4, suggests that SARS-CoV-2 does not use DPP4 for viral cell entry [23,68,69].

Successively, we tested the capacity of neutralization of the SARS-CoV-2 pseudovirus infection by using two different approaches never employed before on human cells. The first one relies on the use of a synthetic tetravalent antibody Fab 15033-7 that binds the SARS-CoV-2 S protein receptor-binding domain (RBD) and blocks its interaction with ACE2. Its extraordinary potency, previously tested on Vero E6 cells [7], has been further confirmed in our hLORG model. These findings support the idea that this human antibody could be used to develop an antiviral drug that blocks the virus from entering the host cells, preventing it from multiplying and causing sickness. Antibodies have a lengthy serum half-life and are frequently well tolerated, particularly when based on human frameworks [7]. In addition to their potential therapeutic utility, anti-SARS-CoV-2 specific nAbs are required for the development of diagnostic assays.

Based on the involvement of DPP4 in SARS CoV2 entry [10], we also tested the inhibitory effect of a DPP4 peptide in our in vitro model of hLORGs. Recently, Murdocca and collaborators [11] have selected a synthetic peptide, DPP4_270–295_, showing a higher binding affinity (HADDOCK score value between −84.3 and −95.3) for the S protein variants that contain mutations in the RBD. Data have demonstrated that DPP4_270–295_ acts in order to hinder the virus in recognizing the DPP4 receptor. As a result, the virus’s ability to enter host cells is inhibited. Our research for the first time demonstrated that employing antiviral peptides against all SARS-CoV-2 variants in a hLORGs model is an effective therapeutic strategy.

Moreover, a SARS-CoV-2 infection stimulates the expression of interferons, cytokines, and chemokines, as well as activating important inflammasome pathway genes, according to previous research [16]. Our findings demonstrated that the expression patterns of genes implicated in innate immunity and inflammation upregulated in response to the hLORGs infection. Both monoclonal antibodies and synthetic peptides evaluated in this study were demonstrated to be able to shut down this inflammatory and cytokine immunitary burst. Therefore, both methods can be used to modify specific protein interactions able to induce an in vivo humoral response or other signaling pathways [70]. Previously, Tiwari and colleagues reported that LORGs infected by SARS-CoV-2 triggered genes of inflammasome pathways (such as *NLRP3, NLRC4, ASC, IL-18,* and *caspase-1*). This leads to an increase in lung inflammation and cell death [16,71].

The dynamics of the connection between DPP4/CD26 localization and site inflammation of the lungs, due to COVID-19, appeared to be verified [59]. Recent evidence has shown that DPP4 plays a key role as a co-receptor and modulator in the entry mechanism and aggressiveness of SARS-Cov-2 in target tissues [59,72], and that DPP4 inhibition could antagonize this mechanism. Here, we demonstrated a strong reduction of inflammasome pathways after neutralization of the infection by Fab IgG 15033-7 or DPP4 peptide treatment. Data have been obtained evaluating gene expression, and further experiments will be performed on the protein profiles of the markers.

Altogether, these data advance our understanding of the pathogenesis of the COVID-19 disease, evidencing potential therapeutic treatments focused on virus neutralization that is able to prevent virus loading and reduce inflammation and lung damage.

Very recently, a novel variant, called Omicron or B.1.1.529, has been identified in 149 countries across all six WHO Regions [73]. There is convincing evidence that Omicron has a significant growth advantage over Delta and is thus becoming dominant all over the world. To date, it has been detected in around 71.5 percent of infected patients solely in the United Kingdom [74,75]. Thus, upcoming studies will be needed to test both monoclonal antibodies and synthetic peptides on these novel variants, in order to verify their efficacy. Moreover, in vitro studies using actual SAR-CoV-2 strains will be conducted to confirm our data obtained on safer pseudotype viruses, even if by now the latter are routinely used for studying the SARS-CoV-2 mechanism of infection.

## 5. Limitation of the Study

An important drawback in using hLORGs derived from hiPSCs is their heterogeneity with respect to maturation, even when using optimized protocols. In our study, we have assessed the expression of the markers of surfactant protein B and C, even if their ratio is not comparable to that reported in literature. Moreover, the expression of the genes encoding for proteins known to influence SARS-CoV-2 entry (*ACE2*, *DPP4*, *CD147*, *PCSK3*, and *TMPRSS2*) has been shown [76]. The expression levels are evidently lower than those from adult human lung tissue, and thus further improvements are necessary to bridge the gap towards mature alveolar epithelial cells. This aspect can be due to several factors, including the absence in vitro of supporting cells during the differentiation process (i.e., the surrounding endothelial cells allowing efficient gas transfer). Moreover, lung tissue in vivo is exposed to the mechanical forces of breathing that, stretching the alveolar cells, can contribute to maturation and surfactant secretion. Moreover, this aspect, which provides a more realistic alveolar environment, is missing in in vitro culture, reducing the possibility of obtaining a homogenous population [77]. In any case, the hLORGs obtained in this study are responsive to infection with SARS-Cov2, expressing markers of the multistep SARS-CoV-2 entry process and mirroring features of SARS-CoV-2 infection (robust induction of chemokines) [76].

Most importantly, the detection of lamellar bodies confirmed the presence of relatively mature type 2 pneumocytes.

## Supplementary Materials

The following are available online at https://www.mdpi.com/article/10.3390/cells11071235/s1, Figure S1: Schematic for differentiation from hiPSCs to Lung Organoids (hLORGs); Figure S2: RT-qPCR analyses of *SFTPB* and *SFTPC* gene in hLORGs; Figure S3: RT–qPCR analyses of proteins involved in SARS-CoV-2 entry; Figure S4: Histological analysis of hLORGs infected with pseudo-SARS-CoV-2S; Figure S5: Electron microscopy images of hLORGs infected with pseudo-SARS-CoV-2S.
