# Supplementary material for: Two Different Therapeutic Approaches for SARS-CoV-2 in hiPSCs-Derived Lung Organoids
Source: Cells. 2022 Apr 5;11(7):1235. doi: 10.3390/cells11071235 (PMC8997767; doi:10.3390/cells11071235)
Supplement: Supplementary file 1 [file cells-11-01235-s001.zip › cells-1577026-supplementary.pdf]

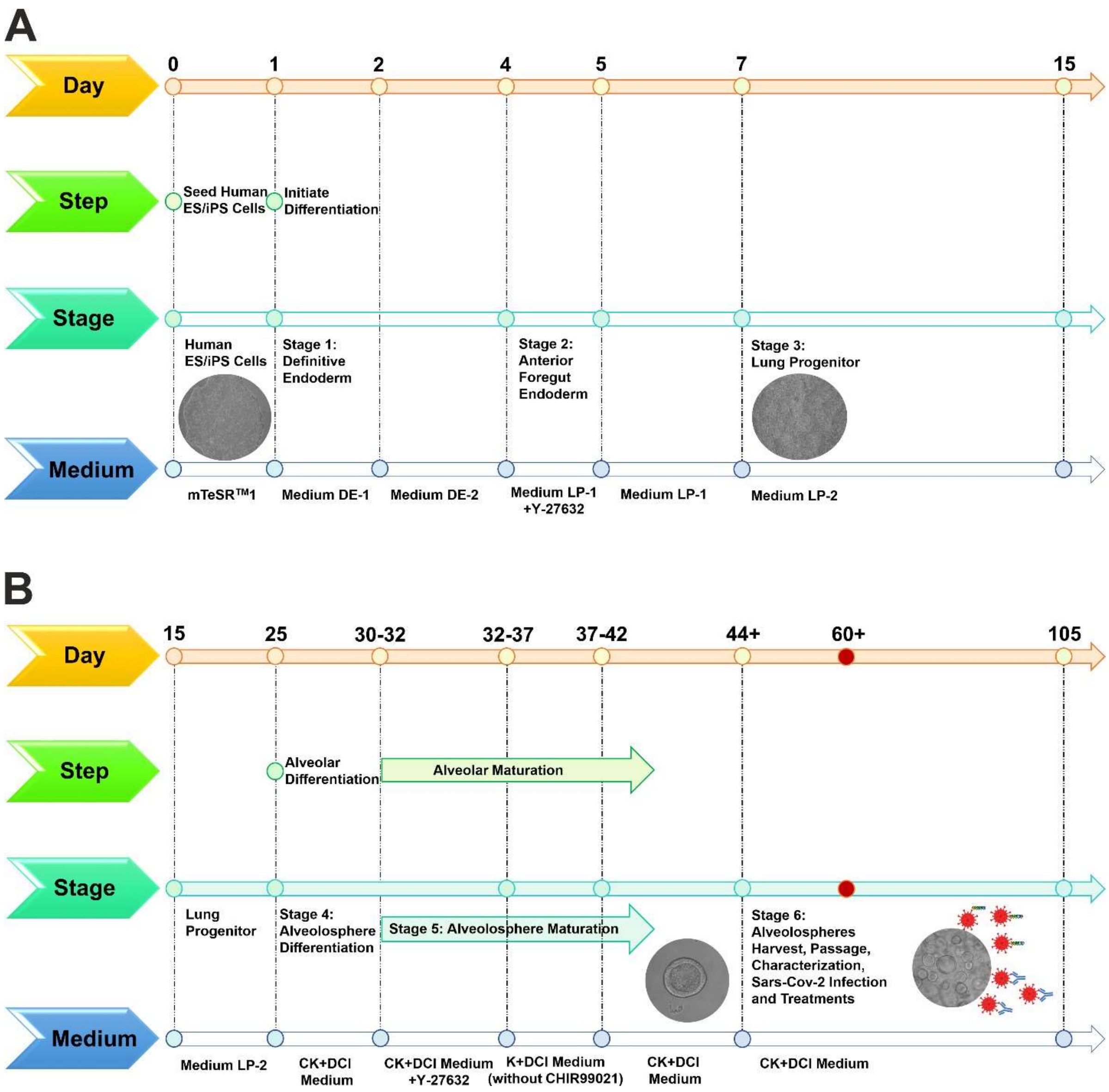

Figure S1. Schematic for differentiation from hiPSCs to Lung Organoids (HLORGs) (**A**) Flowchart protocol of hiPSCs differentiation to lung progenitors using StemDiff Lung Progenitor kit. (**B**) Workflow for lung progenitors into hLORGs: after characterization, hLORGs were infected and treated with Fab-IgG 15033-7 or $DPP4_{270\text{-}295}$ between days 60-67. The 3D cultures were maintained until day 105.

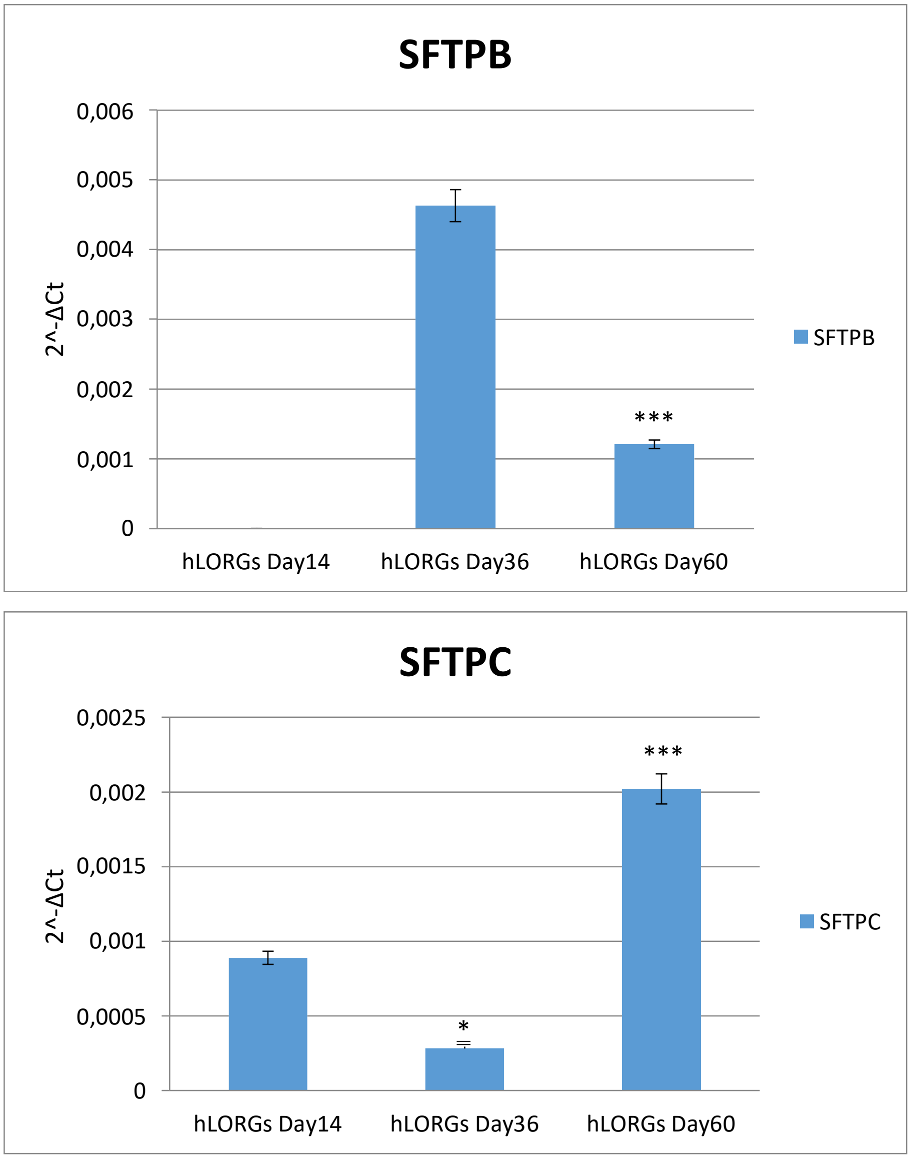

Figure S2. RT–qPCR analyses of *SFTPB* and *SFTPC* genes in hLORGs at days 14, 36 and 60 of differentiation. Data are reported as absolute values after normalization versus a housekeeping gene. The transcript SFTPB/C ratio is about 16: 1. Data are representative of three independent experiments and reported as mean ± SD. (*p<0.05;***p<0.001 by one-way ANOVA test).

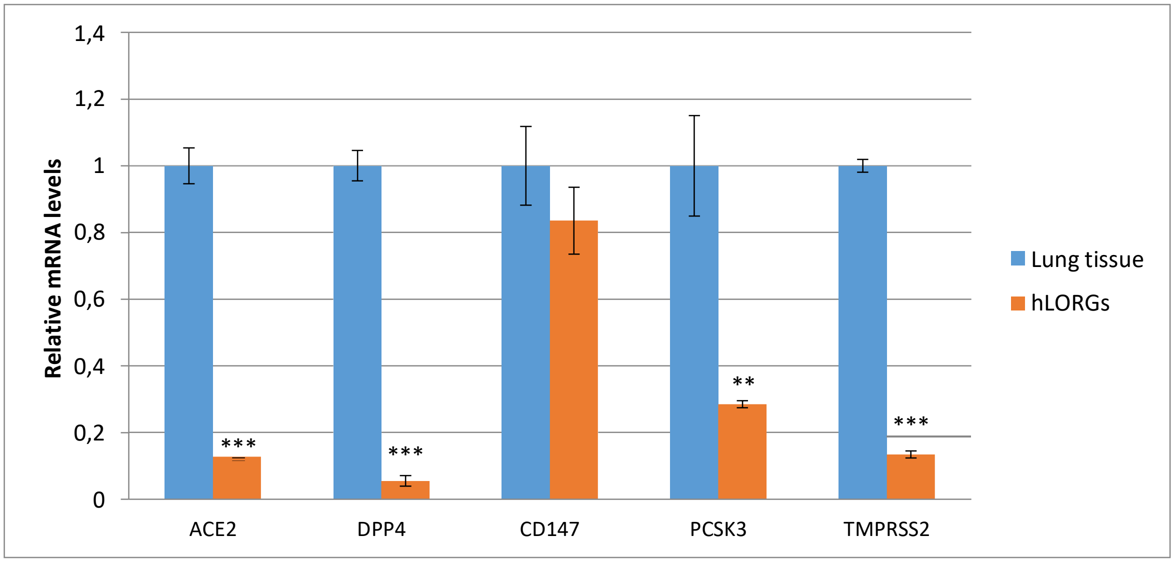

Figure S3. RT–qPCR analyses of proteins involved in SARS-CoV-2 entry (ACE2, DPP4, CD147, PCSK3, TMPRSS2) in hLORGs and human lung tissue. Data are representative of three independent experiments and reported as mean ± SD. **p<0.01,***p<0.001 by one way ANOVA test.

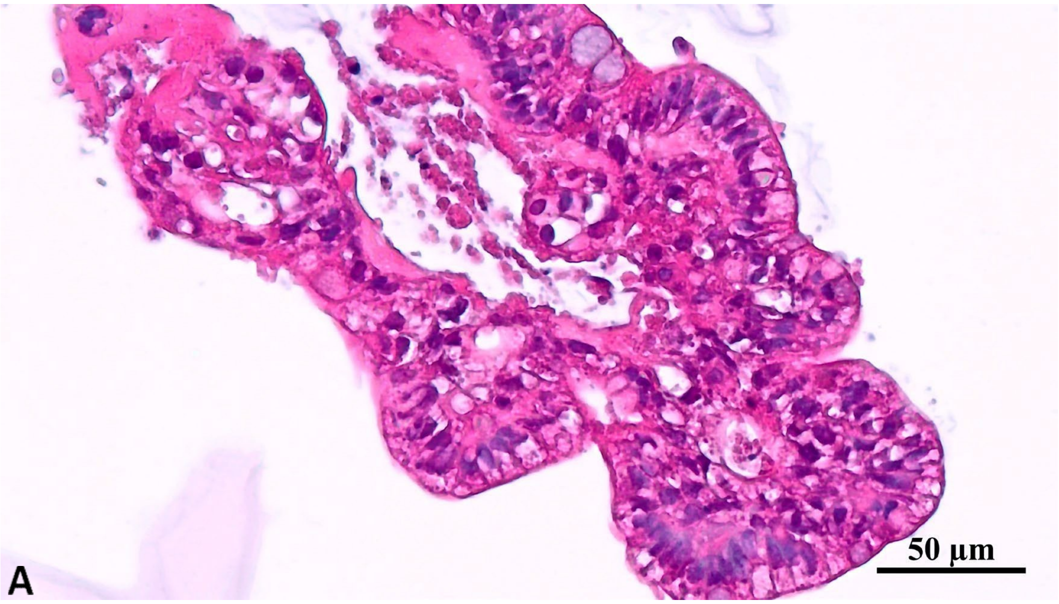

A

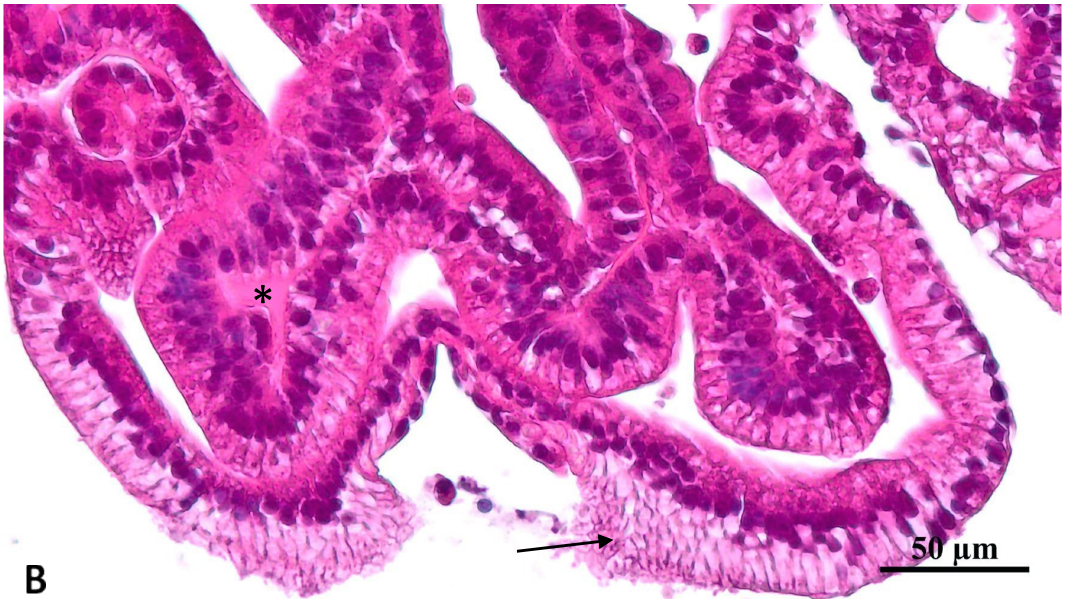

B

Figure S4. Histological analysis of alveolocytes infected or not with pseudo-SARS-CoV-2S (**A, B**) Haematoxylin and eosin-stained sections of infected or not alveolopheres with **pseudo-SARS-CoV-2S** (original magnification, 200X). Representative images show cellular damages of infected alveolocytes that display hyperplasia (arrow) with enlarged nuclei and prominent nucleoli with hyaline deposition (pink deposits, asterisk).

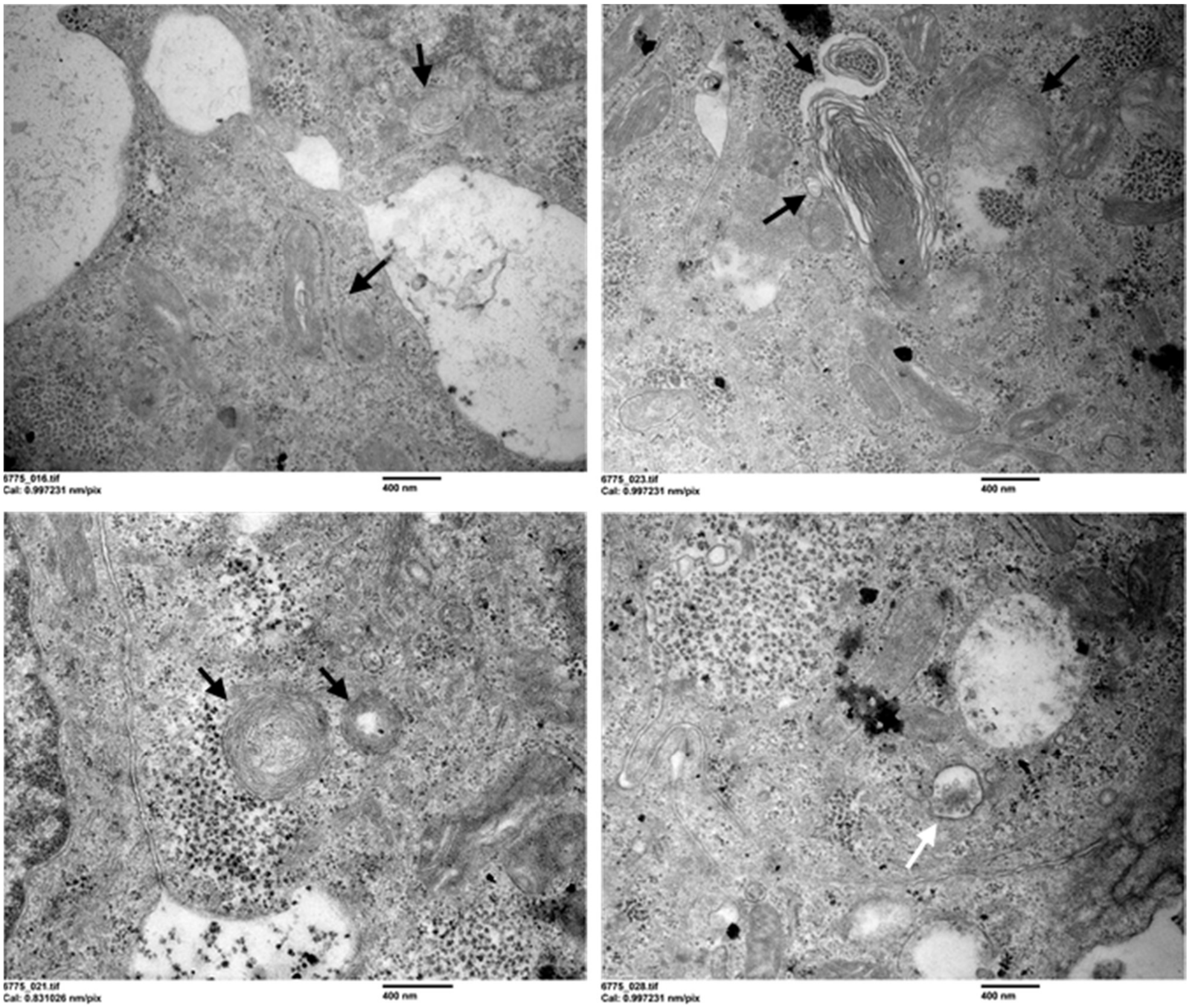

Figure S5. Ultrastructural analysis of hLORGs infected with pseudo-SARS-CoV-2S. TEM images of hLORGs (48 hours post-infection) show the presence of intracytoplasmic lamellar bodies (black arrows) and viral particles within a single membrane vesicle (white arrow).
